# Localized circumferential narrowed bronchial wall lesions in allergic bronchopulmonary aspergillosis

**DOI:** 10.1002/rcr2.612

**Published:** 2020-07-07

**Authors:** Yurina Murakami, Yoshihiro Kitahara, Tomohiro Uto, Jun Sato, Shiro Imokawa, Takafumi Suda

**Affiliations:** ^1^ Division of Respiratory Medicine Iwata City Hospital Shizuoka Japan; ^2^ Second Division, Department of Internal Medicine Hamamatsu University School of Medicine Hamamatsu Japan

**Keywords:** *Aspergillus fumigatus*, bronchoscopic findings, chest CT findings, mucoid impaction, tracheobronchial lesions

## Abstract

We present a 67‐year‐old man with allergic bronchopulmonary aspergillosis (ABPA), whose chest computed tomography (CT) scans showed localized circumferential bronchial wall thickening in the right main bronchus to middle truncus. Chest CT scans showed high‐attenuation mucus in the right B^8^ bronchus. Bronchoscopy showed narrowing of the right main bronchus and inspissated mucus plugging in the right B^8^ bronchus. Histological examination of the right main bronchus revealed bronchial inflammation with numerous neutrophils and plasma cells. Treatment with corticosteroid and antifungal agents resulted in resolution of the symptoms and right bronchial lesions. The clinical course may indicate that the bronchial lesions were associated with ABPA.

## Graphical abstract

The typical bronchial manifestations of allergic bronchopulmonary aspergillosis (ABPA) include intraluminal retention of inspissated mucus plugging and chronic inflammation of airway walls with eosinophils, lymphocytes, and neutrophils. Bronchiectasis and bronchial wall thickening can be seen on chest computed tomography (CT) scans. However, localized circumferential narrowing of the bronchus has not been reported. Herein, we report such a patient with ABPA, whose bronchial lesions disappeared after treatment with prednisolone and antifungal agents.

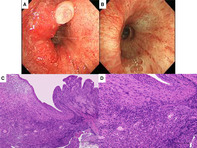

## Introduction

The typical bronchial manifestations of allergic bronchopulmonary aspergillosis (ABPA) include intraluminal retention of inspissated mucus plugging and chronic inflammation of airway walls with eosinophils, lymphocytes, and neutrophils [1]. Bronchiectasis and bronchial wall thickening can be seen on chest computed tomography (CT) scans [1]. However, localized circumferential narrowing of the bronchus has not been reported. Herein, we report such a patient with ABPA, whose bronchial lesions disappeared after treatment with prednisolone and antifungal agents.

## Case Report

**Figure 1:**
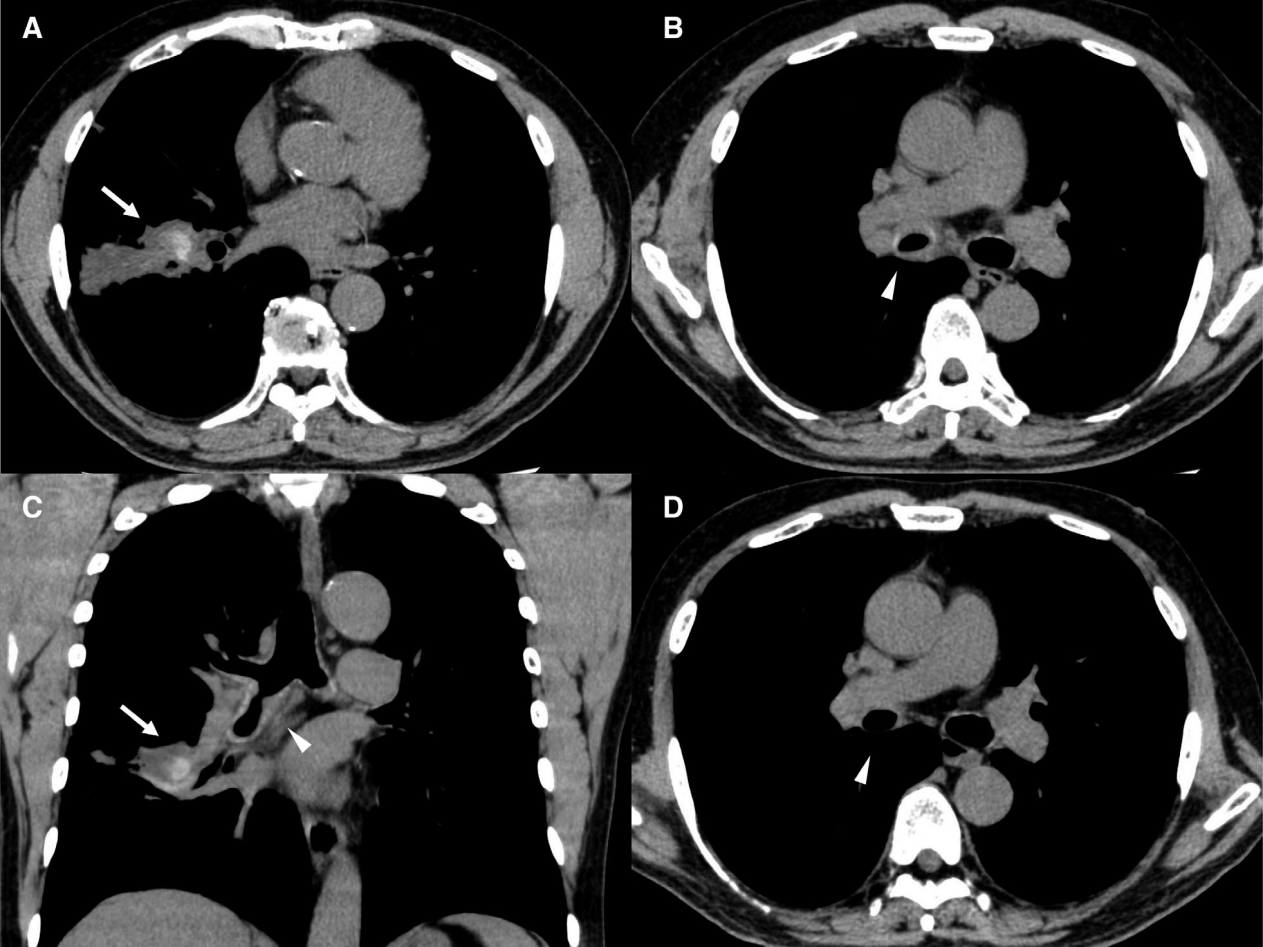
(A) Chest computed tomography (CT) findings (soft tissue window) showing highly attenuated, mucus‐filled, dilated bronchi in the right lower lobes (arrow). (B) Chest CT findings (soft tissue window) showing narrowing of the right main bronchus to middle truncus (arrowhead). (C) Coronal image of chest CT findings (soft tissue window) showing narrowing of the right main bronchus to middle truncus (arrowhead) and high‐attenuation mucus in the right B^8^ bronchus (arrow). (D) Chest CT findings (soft tissue window) after two months of treatment with systemic corticosteroid and antifungal agents showing resolution of the right tracheobronchial lesions (arrowhead). Prednisolone was reduced to 20 mg/day and the dose of voriconazole was 200 mg/day.

**Figure 2:**
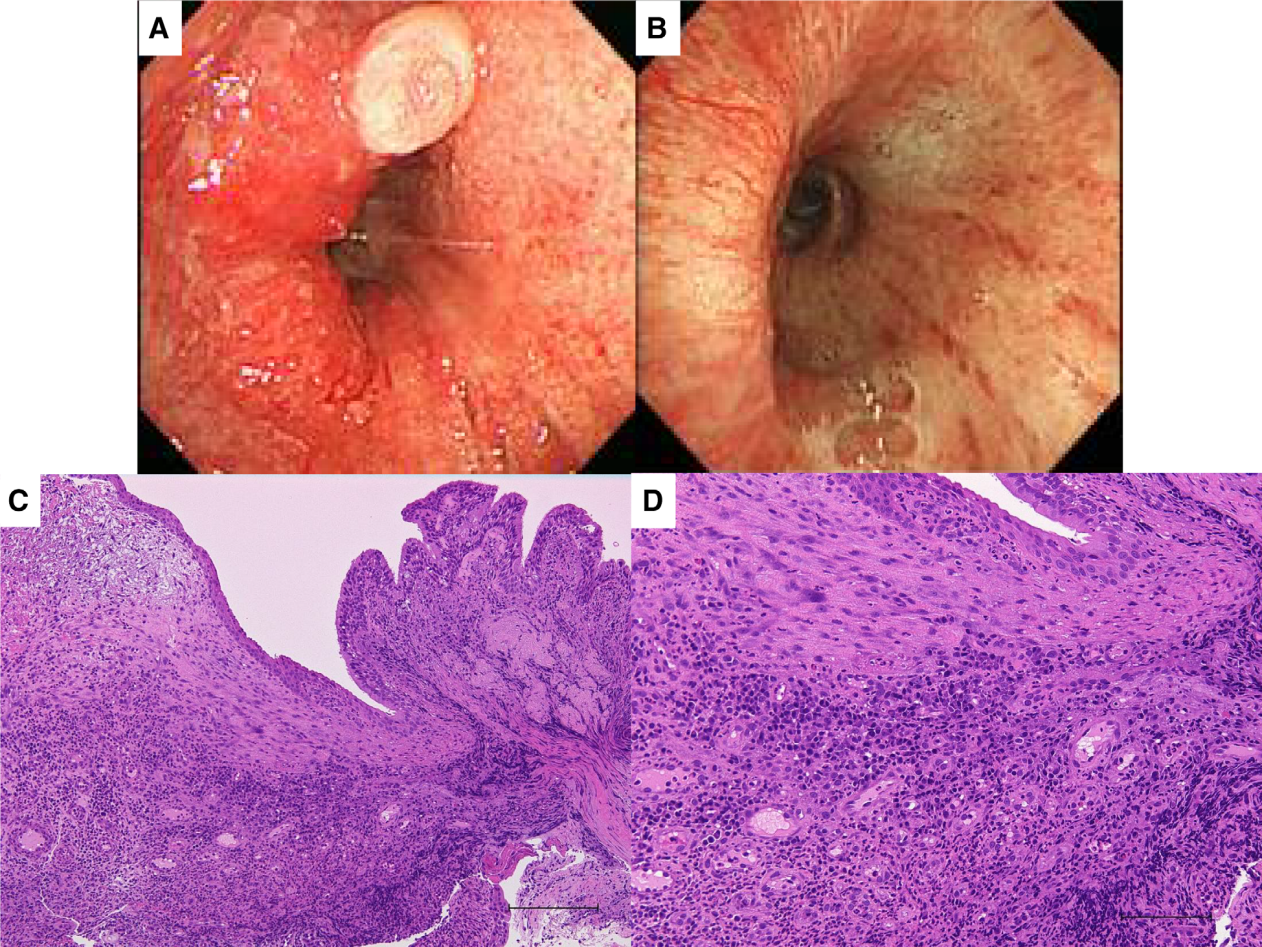
(A) Fibreoptic bronchoscopic findings showing narrowing of the right main bronchus to middle truncus with oedematous, reddened polypoid mucosal lesions. (B) Fibreoptic bronchoscopic findings after two months of treatment with systemic corticosteroid and antifungal agents showing the resolution of the localized narrowed bronchial mucosal lesions. (C) Biopsy specimens of the mucosal lesions showing subepithelial and submucosal oedematous changes with inflammatory cell infiltration. Bar indicates 500 μm. (D) The inflammatory cells included plasma cells and neutrophils. There were few eosinophils. Bar indicates 100 μm.

A 67‐year‐old man was referred for evaluation of cough and sputum, which appeared three months prior to the visit to our hospital. He had an allergic rhinitis and a 15‐year history of bronchial asthma that had been diagnosed at previous hospital based on recurrent wheezing episodes. He had been treated with fluticasone 750 μg/day and formoterol 30 μg/day, but his symptoms continued for three months. Spirometry revealed forced expiratory volume in 1 sec (FEV_1_)/forced vital capacity (FVC) of 70.1% and FEV_1_ of 2.41 L (91.3% of predicted). The fraction of exhaled nitric oxide was 48 ppb. Chest CT scans showed highly attenuated, mucus‐filled, dilated bronchi in the right B^8^ bronchus. Eosinophil count was 943.5/μL and total immunoglobulin E (IgE) level was 1111 IU/mL. *Aspergillus*‐specific IgE and precipitating antibody were positive. Bronchoscopic findings showed intraluminal retention of inspissated mucoid secretions in the right B^8^ bronchus. Histological examinations of the mucoid secretions revealed clusters of degenerating eosinophils alternating with relatively acellular mucin, compatible with mucoid impaction. The diagnosis of ABPA was made based on the International Society for Human and Animal Mycology criteria (positive for predisposing conditions: asthma; positive for both the obligatory criteria: total baseline serum IgE level > 1000 IU/mL and elevated *Aspergillus‐*specific IgE; and positive for all the three other criteria: eosinophilia >500/μL, serum precipitating antibody to *Aspergillus fumigatus*, and consisting radiological opacities) [2]. Treatment with systemic corticosteroid and itraconazole was recommended, but he refused this therapy. Thus, conservative follow‐up was undertaken. One month later, he experienced mild asthma attacks and needed short‐acting beta‐agonist at several times, which improved his symptoms transiently. Six months later, he was referred to our hospital with a two‐week history of progressive productive cough and low‐grade fever. Chest CT scans showed worsening of the high‐attenuation mucus in the right B^8^ dilated bronchus (Fig. 1A, C) with the appearance of localized circumferential wall thickening in the orifice of the right main bronchus (Fig. 1B, C). The latter finding was not present at the time of ABPA diagnosis. Eosinophil count was 676/μL and total IgE level was 1599 IU/mL. Spirometry revealed FEV_1_/FVC of 67.6%, FEV_1_ of 2.25 L (84.6% of predicted), and peak expiratory flow of 5.57 L/min (67.3% of predicted). Bronchoscopy showed narrowing of the right bronchus (Fig. 2A) and histopathological examination revealed bronchial inflammation with neutrophils and plasma cells (Fig. 2C, D). Tissue culture of the lesion was negative for *A. fumigatus*. Mucoid impaction was noted in the right B^8^ bronchus and tissue culture of the mucoid impaction grew *A. fumigatus*. Histological examination of the bronchial mucosa adjacent to the mucoid impaction showed inflammation with numerous eosinophils. Immediate and late skin reactions for *Aspergillus* yielded positive results. Treatment with systemic corticosteroid (prednisolone 40 mg/day: 0.5 mg/kg/day) and itraconazole (400 mg/day), the latter was switched to voriconazole (960 mg/day of loading dose and 600 mg/day of maintenance dose) because of liver dysfunction and rash, resulted in resolution of the symptoms and right bronchial lesions (Figs. 1D, 2B). Voriconazole was reduced to 200 mg/day based on the serum concentrations of it. Prednisolone was gradually tapered off for 16 weeks and the duration of antifungal treatments was 16 weeks. Total IgE level decreased from 1599 to 167 IU/mL after these treatments.

## Discussion

Chest CT findings in patients with ABPA usually show bronchiectasis, bronchial wall thickening, and mucus plugging [1]. Occasionally, atelectasis, lobar collapse, and nodules can be seen, and mucus plugging can manifest a “finger‐in glove” appearance as a result of impacted mucus along the airway and its branches [1]. However, localized circumferential narrowing of the bronchus is extremely rare. In our case, bronchoscopic findings showed narrowing of the right main bronchus to middle truncus with oedematous, reddened polypoid mucosal lesions. These bronchial lesions improved dramatically after treatment with prednisolone and antifungal agents. The clinical course may indicate that the bronchial lesions were associated with ABPA.

Histological findings of the narrowed bronchial lesions showed subepithelial and submucosal oedematous change with inflammatory cell infiltrations involving numerous plasma cells and neutrophils. These findings differed from those of the bronchial mucosa adjacent to the mucoid impaction at the distal aspects of the bronchi, which showed numerous eosinophils and lymphocytes. The mechanisms for these different pathological findings are unclear. We speculate that many factors may have contributed to the differences in these bronchial lesions, such as the local and systemic immune statuses and alterations to host tissues as well as *Aspergillus* proteolytic enzymes, interleukin (IL)‐8‐mediated neutrophilic inflammation, and Th2 responses to *Aspergillus* antigens in the asthmatic milieu [3, 4, 5].

There are four main clinical categories of *Aspergillus*‐related respiratory disease [3]: saprophytic infections (aspergilloma), allergic pulmonary diseases (ABPA), invasive disease (invasive and subacute invasive bronchopulmonary aspergillosis), and toxic reactions [3]. On rare occasions, one distinct *Aspergillus*‐related entity can change into another; for example, an aspergilloma may progress to semiacute invasive or invasive pulmonary aspergillosis [3]. Meanwhile, the line between saprophytic and invasive *Aspergillus* infections is not well defined, and borderline cases can be expected in clinical practice [3]. Combined forms of tracheobronchial aspergillosis and ABPA have also been reported [5]. The present case raises the possibility that the two clinical entities of ABPA and saprophytic infection may exist, but *Aspergillus* was not detected by histological examination or culture of tissue samples from the narrowed endobronchial lesions.

In conclusion, we present here a patient with ABPA associated with localized circumferential narrowing of the bronchus away from the mucoid impaction. The pathogenesis and the optimal therapy are unclear and further investigations are needed to clarify them.

### Disclosure Statement

Appropriate written informed consent was obtained for publication of this case report and accompanying images.
